# Rodent models for oral microbiome research: considerations and challenges- a mini review

**DOI:** 10.3389/froh.2024.1439091

**Published:** 2024-10-01

**Authors:** Divya Gopinath, Deepak Pandiar, Zhengrui Li, Swagatika Panda

**Affiliations:** ^1^ Basic Medical and Dental Sciences Department, College of Dentistry, Ajman University, Ajman, United Arab Emirates; ^2^ Centre of Medical and Bio-Allied Health Sciences Research, Ajman University, Ajman, United Arab Emirates; ^3^ Department of Oral Pathology and Microbiology, Saveetha Dental College and Hospitals, Saveetha Institute of Medical and Technical Sciences, Saveetha University, Chennai, India; ^4^ Department of Oral and Maxillofacial-Head and Neck Oncology, Shanghai Ninth People’s Hospital, Shanghai Jiao Tong University School of Medicine, Shanghai, China; ^5^ College of Stomatology, Shanghai Jiao Tong University, Shanghai, China; ^6^ National Center for Stomatology, Shanghai, China; ^7^ National Clinical Research Center for Oral Diseases, Shanghai, China; ^8^ Shanghai Key Laboratory of Stomatology, Shanghai, China; ^9^ Shanghai Research Institute of Stomatology, Shanghai, China; ^10^ Shanghai Center of Head and Neck Oncology Clinical and Translational Science, Shanghai, China; ^11^ Department of Oral Pathology and Microbiology, Institute of Dental Sciences, Siksha 'O' Anusandhan University, Bhubaneswar, India

**Keywords:** oral, microbiome, microbiota, mice: mouse: rodents: animals, *in vivo*

## Abstract

Rodent models have been commonly employed in oral microbiota research to investigate the relationship between bacteria and oral disease. Nevertheless, to apply the knowledge acquired from studies conducted on rodents to a human context, it is crucial to consider the significant spatial and temporal parallels and differences between the oral microbiota of mice and humans. Initially, we outline the comparative physiology and microbiology of the oral cavity of rodents and humans. Additionally, we highlight the strong correlation between the oral microbiome of rodents and genetic makeup, which is influenced by factors including vendor, husbandry practices, and environmental conditions. All of these factors potentially impact the replicability of studies on rodent microbiota and the resulting conclusions. Next, we direct our attention toward the diversity in the microbiome within mice models of disease and highlight the diversity that may potentially affect the characteristics of diseases and, in turn, alter the ability to replicate research findings and apply them to real-world situations. Furthermore, we explore the practicality of oral microbial models for complex oral microbial diseases in future investigations by examining the concept of gnotobiotic and germ-free mouse models. Finally, we stress the importance of investigating suitable techniques for characterizing and managing genetically modified organisms. Future research should consider these aspects to improve oral microbiome research's translational potential.

## 1 Introduction

The use of mice and rats as a model has been crucial in understanding the causes, development, and potential treatments for several human diseases. Obtaining consistent results from the model was challenging for a long time due to the presence of inconsistencies in nutrition, infection, age of animals, housing, and several other factors. Although numerous research cited in the literature have provided new insights into several human diseases, it is common for them to be deficient in sufficient information, limiting their translational value. While many studies mentioned in the literature are clear, it is also not uncommon for them to lack enough information to make judgment easier (1).

The microbiome is the collective genetic material of all the microorganisms that inhabitate on and inside human body, including bacteria, protozoans, fungi and viruses. Under unfavorable conditions, few microbes have been shown to become pathogenic and may result in dental caries, periodontitis, abscesses, endocarditis, and various oral potentially malignant disorders/invasive neoplasms. Microorganisms of any type may result in or trigger the pathogenic process; while some are established conditions, the role of others is questionable. For instance, candida (or candidiasis) had always been considered carcinogenic (2), but their definitive role has now been deemed contentious to the latest World Health Organization updates of head and neck tumors in 2022 (3). Previous studies have repeatedly indicated a spatial role of the oral microbiome in the tumorigenesis of oral cancer (4). The role of periodontal diseases has been positively correlated with an increased risk of oral potentially malignant disorders (OPMDs) (5) and oral squamous cell carcinoma (OSCC) (6), which led the research to hypothesize that the inflammatory microbiome of periodontitis may also play a plausible role in development and progression of oral cancer. The need for an ideal animal model is thus deemed necessary for the duplication of these diseases and for basic, translational research. Rodent models have been used to study oral microbiomes and diseases associated with microbial dysbiosis. The chief advantages of the application of rodents for experimental procedures include ease of breeding and handling, comparatively lower maintenance cost, availability, and an adequately sized oral cavity for easy inoculation (and sample collection). Although rodents and humans share numerous morphological, histological, and physiologic characteristics, there are significant disparities in oral microbiome. Therefore, it is not unexpected that there are substantial variations in the composition of the gut microbiota, both in terms of the types of microorganisms present and their relative abundance. Research has demonstrated significant variations in microbial composition among different breeds of mice (7). Given these findings, it is natural to question using mouse models for extrapolating the oral microbiome to humans. One of the possible explanations is that there is currently no superior option. Here, we review the physiology and oral microbiology of the rodents in detail. Further, we focus on the mice models tried and tested for research involving oral microbiome and highlight the recent advancements in science that could be utilized to improve the oral microbiome's translational potential *in vivo* research.

## 2 Physiology of the oral cavity of mice and rats

The anatomical morphology, physiology, and histological characteristics of the oral cavity of rodents, one of the most typical animal models, resembles the human rima oris (8). Rodents (order Rodentia), the largest mammalian group with extreme diversification, comprises 40% of all the mammal species of kingdom Animalia and are subclassified into three major suborders based on the anatomical variations and functional differences in masseter muscles (8, 9). All the rat-like/mice-like rodents are categorized as *Myomorpha.* The other two suborders are *Caviomorpha/Hystrichomorpha* and *Sciuromorpha* (9).

**Figure 1:**
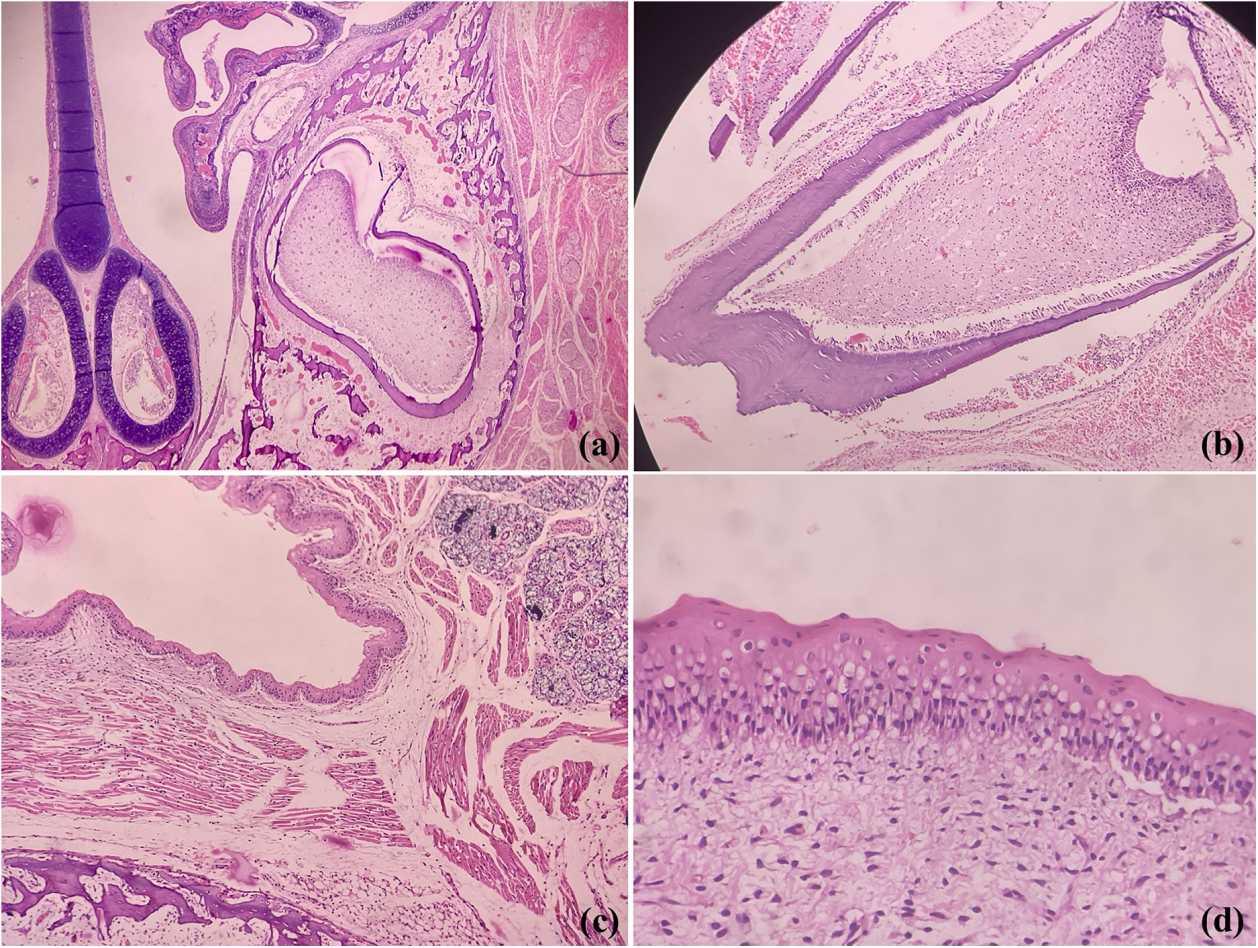
Photomicrographs of H&E stained sections showing coronal section of rodent face demonstrating developing tooth germs, maxillary sinus and nasal septum **(a)**, developing molar **(b)**, fetal epithelium. salivary gland tissue, bone and skeletal muscles **(c)** and oral epithelium **(d)** (Picture courtesy Dr Suganya Panneer Selvam, Oral Biologist, Saveetha Dental College and Hospitals).

In contrast to human dentition (2/2I, 1/1C, 2/2PM, 3/3M), who have well-formed crowns and roots of all teeth (32 permanent), all rodents species have elodont aradicular incisors, lack the canines and bears a long space between incisors and ‘cheek-teeth’-premolars and molars (9). It should be noted that the rats and golden hamsters also lack the premolars, yielding a dental formula as 1/1I, 0/0 C, 0/0 P and 3/3M (total 16 teeth). However, odontogenesis is akin to human tooth development involving the same types of cells, making these a perfect model for analyzing odontogenic lesions. Figure 1 shows photomicrographs of developing rat tooth germs and adjacent oral structures where we can find close resemblances with human tooth germs.

Further, the differences lie in the jaws, arrangement of the masseter, and zygomatic arch (10, 11). In contrast to humans, where the jaw movements are restricted to complex arrangement and synchronization of muscles at temporomandibular joints (TMJ), the few species of rats show unossified cartilaginous tissue at mandibular symphysis area, allows specific movements between the mandibular bones. The symphysis in human mandibles is fused with no movements. Three distinct layers have been identified viz. superficial masseter, deep masseter and zygomatico mandibularis (9), contrariwise, only two sets of fibres are seen in human, the superficial and deep. The similar angulation fibers and function of these strong muscles of mastication allow easy recapitulation of temporomandibular joint movement and disorders in animal models.

**Figure 2:**
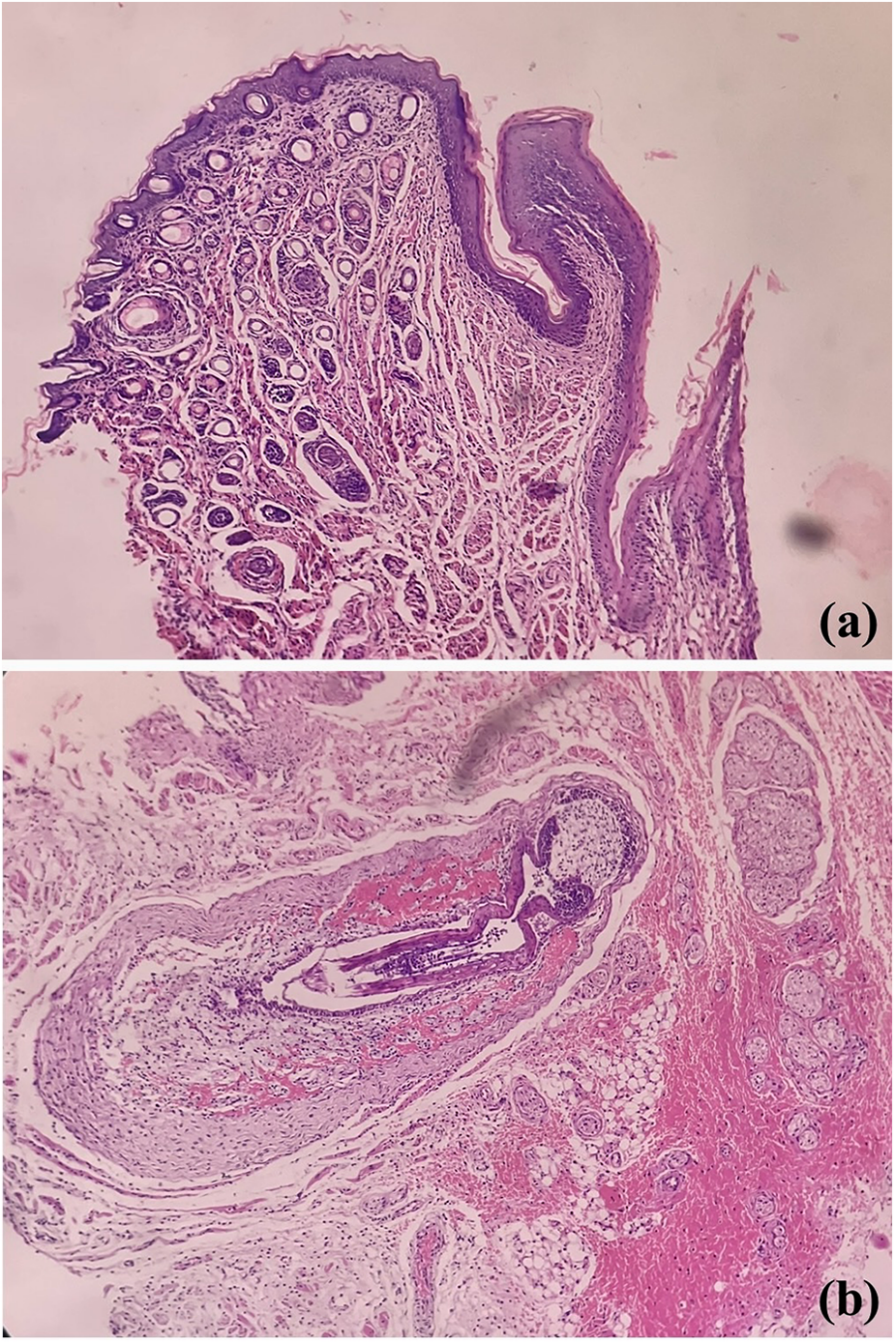
Photomicrographs of H&E stained sections showing labial orthokeratinized stratified squamous epithelium and numerous dermal adnexal structures **(a)** and developing hair follicles [vibrissae, **(b)**].

Pertaining lining epithelium in rats, which serves as the prime oral structure for analysis of oral microbiota and carcinogenesis, shows many similarities with the oral mucosa of the human mouth. Externally delineated by lips, labial skin which is orthokeratinized stratified squamous cell type contains abundant hair, adnexa, and numerous vibrissae, structures lacking in human (Figure 2). Further, unlike us, a cleft is noted between upper incisors; thus, the labial frenulum is not noted. The lingual frenum is also not seen in rats (12). The similar stratification of the epithelia is noted; however, the entire oral cavity is variably ortho-keratinized. The human oral cavity demonstrates the transition from keratinized labial mucosa to non-keratinization in the buccal and floor of mouth region to para- to ortho-keratinization on attached gingival or palatal mucosa. The tongue is specialized with thousands of taste buds; in contrast, the majority of taste buds are seen on the soft palate in rats, with missing uvula (8). However whether similar physiology, odontogenesis, and palatogenesis between human and rodent oral cavity have an influence on the oral microbial colonisation is not known.

## 3 Oral microbiome of rodents

The bacteria flora of the oral cavity of humans is dominated by the phyla Fusobacteria, Actinobacteria, Firmicutes, Bacteroidetes, Proteobacteria, and Spirochaetes, accounting for approximately 96% of the species detected (13). The normal human flora begins to develop postpartum; an infant is first exposed to microorganisms during birth (13). While the human gastrointestinal tract contributes a significant number of microbiota, the human oral cavity contains as high as 700 different species (13). The bulk of microbes in the oral cavity of rodents were reported to be attributed to the phyla *Firmicutes* and *Proteobacteria*, with most of them belonging to the class *Gammaproteobacteria* (14). The oral microbial communities in humans are more intricate than those in mice, with less than 50% of the oral microbiota in human samples comprising the top ten bacterial species/phylotypes in mice (15). A study revealed that only 27 types of mouth bacteria were common to both mice and humans (15). The earliest communities seen in the murine oral mucosa are similar to those found on the murine skin, indicating that the transmission of these communities likely occurs from the mother's skin during feeding (14). *Staphylococcus sp., Streptococcus sp., Lactobacillus sp., Enterobacteriaceae sp.,* and *Enterococcus sp*. were shown to constitute the main species in the rodents (16).

## 4 Factors that influence oral microbiome composition of experimental rodents

### 4.1 Genetics of the mouse

In murine oral microbiome research, understanding the genetic background of various mouse strains is crucial for ensuring result accuracy and credibility. Studies indicate significant differences in the diversity and richness of the oral microbiomes among mouse strains, such as C57BL/6 or BALB/c (17, 18). These differences significantly affect research into the interactions between microbiota and host health, also challenging model selection and study design. Specifically, the notable differences in types and quantities of oral microbiota across mouse strains are crucial for autoimmune disease research. This underscores the importance of selecting the appropriate mouse strain for a deeper insight into the microbiome's role in specific disease models. Furthermore, research that identifies a direct link between specific genetic markers and the mouse oral microbiome composition enhances our understanding of microbiota-host interactions and offers a scientific basis for selecting suitable mouse models (19, 20). Researchers discovered that, despite identical genetic manipulations, different mouse strains show significant differences in oral microbiome composition when replicating the same pathological changes (21, 22). Further, it has been identified to have a more significant impact than the animal's gender in microbiome studies (23). Prior research has also demonstrated that mice obtained from various suppliers possess unique microbial communities in their oral, stomach, and fecal regions (24).These findings highlight the importance of accounting for the genetic background of mice in microbiome studies.

### 4.2 Diet

Research consistently shows that dietary changes significantly impact mice's oral microbiome composition and diversity. Specifically, high-fat and sugar diets increase certain pathogenic bacteria in the oral cavity, whereas fiber-rich diets enhance microbial diversity, benefiting oral health (25). Studies using mouse models reveal that dietary changes affect the microbiome's composition and are closely linked to oral health status (26). For example, certain dietary habits can promote dental caries and periodontal disease development, closely associated with diet-induced changes in the oral microbiome. Adjusting mice's dietary composition allows researchers to explore the relationships between specific microbial communities and various physiological and pathological states. This approach improves our understanding of microbes’ impact on oral and overall health and how dietary interventions can enhance health. Diet significantly impacts mouse models’ oral microbiome composition; adjusting it is crucial for exploring the relationships between the oral microbiome and health states.

### 4.3 Housing during experiments

Laboratory environmental conditions, namely temperature, humidity, and housing density, significantly affect the oral microbiome composition in mice (27–29). These environmental factors can directly impact the oral microbiome's stability and indirectly alter microbial community balances by affecting mice's physiological states, immune responses, and dietary preferences (30). Extrinsic factors in the environment, such as the shedding of skin or dust particles from caretakers and scientists who handle the mice, as well as the pH of the water, water treatment and the type of food given, play a crucial role in shaping the microbial colonization in laboratory mice (24, 31, 32). The oral microbiome, in general, is crucial for various physiological and pathological processes and demonstrates high plasticity to environmental changes, highlighting its quick adaptability to surrounding shifts (33). Although existing literature highlights the significant impact of environmental conditions on mice's microbiome, further research is required to elucidate the specific mechanisms and effects on physiological and health statuses.

### 4.4 Stress during experiments

Physiological and psychological stress are key factors affecting the composition of the gut microbiome in mice, potentially exerting effects through changes in immune response mechanisms. Under stress conditions, mice exhibit significant changes in the structure of their microbial communities, including oral mucosa, which could further impact their health and susceptibility to diseases (34, 35). Studies have shown that stress affects the diversity and abundance of microbes, possibly by modulating the activity of the immune system, thereby altering the balance of the microbial community (34, 36, 37). Stress hormone cortisol has been directly implicated in modulating the gene expression profiles of the oral microbiome (38). This underscores the importance of stress management in experimental design, especially in research exploring the relationship between oral microbiota and host health. Therefore, managing and measuring stress factors should be central to scientific research design to ensure the accuracy and comparability of results. Methodological considerations, including the assessment of stress levels, are critical for interpreting results and ensuring their reproducibility and generalizability.

### 4.5 Other related factors

Research shows that a mouse's immune system status, whether immunodeficient or hyperimmune, significantly influences its oral microbiome composition. Immunodeficient mice tend to harbor more pathogenic microbes in their oral cavity, whereas hyperimmune mice may suppress some beneficial microbes (39, 40). The use of antibiotics and other medications significantly impacts mice's oral microbiome. Specifically, antibiotics often reduce microbial diversity and lead to the emergence of antibiotic-resistant strains (41).

## 5 Currently used strains in oral microbiome research

In contemporary oral microbiome research, *in vivo* research employs several rodent strains to unravel the complex host-microbe interactions in infectious oral diseases. The choice of specific strains is guided by the unique characteristics of each strain, which offer unique advantages for specific aspects of these diseases, allowing researchers to examine genetic susceptibility, immune responses, and microbial dynamics.

### 5.1 Caries and apical periodontitis

Previously, Wistar, Sprague-Dawley, and Osborn strains were the most frequently used rat strains in caries research (42). Hsiao et al. used two rat models such as pathogen-free Sprague–Dawley(SD) rats as the Nutritional Microbial Bacterial Model and Sprague–Dawley adult rats as Pulp Disease Induction Models to observe the progress of dental caries and pulp disease (43). Some of the rodent models in caries microbiome research include NFS/N mice strain to determine the caries susceptibility (41), Sprague–Dawley rats for caries induction (42), and BALB/cA and C3H/HeN mice strains for the caries microorganism sensitivity (44). C57BL/6 mice was used to evaluate microorganism mediated pulpitis-induction (18, 44–45). Germ-free adult male C57BL/6 J mice was used recently disparity in metabolic activity and lactic acid generation between monozygotic twins with contrasting caries experiences and their oral microbiome (46). For investigations on microbe-induced apical periodontitis, the most commonly used models include C 57B/L6 mice and (BALB/c) mice (47–49). A recent study utilized apoE−/− mice to establish a *P. gingivalis*-induced chronic apical periodontitis (CAP) model to understand the association of CAP with gut microbiota as well as atherosclerosis by 16S rRNA sequencing and microbial metabolomics (50). Wistar rats were also used to study the effect of *F. nucleatum-induced* apical periodontitis on gut microbiota (51).

### 5.2 Periodontitis

By introducing multiple bacterial species into the model, researchers can mimic the intricate interactions and synergistic effects among different pathogens, the role of host immune responses in shaping the periodontal microbiota and disease progression, the interplay between the host immune system and the oral microbiome in driving periodontal tissue destruction, and assessing the efficacy of treatments in a more realistic disease context, thus providing insights into disease pathogenesis that may be missed in monomicrobial models. In this context, the periodontal inoculation model has been successfully used to evaluate mechanisms of periodontal inflammation, pathogenic differences between different periodontal bacteria, and alveolar bone loss (52, 53). C57BL/6, C57BL/6 J (WT, -TLR2(KO), -TLR4(KO), -TLR2,&4KO) mice, CD1 Swiss mice, C57BL/6 J female mice are some of the strains, and their knockouts that have been used in experimental periodontitis with microbes (54). Klausen has reviewed the studies on periodontitis in rat models and concluded that germ-free Sprague–Dawley(SD) rats were the most commonly used rodent strains, followed by Lewis specific pathogen free (LEW-SPF) and Rowett SPF (ROW-SPF) (55). Major histocompatibility complex (MHC), cytokine, and adhesin molecule knockout mouse strains have also been used to evaluate the role of these mediators in *P. gingivalis*-mediated periodontal tissue destruction (56). The Baker mouse model, chemically induced mouse model, and murine incisor abscess model have been used to study the interaction of monomicrobial infections in apical periodontitis (57). In contrast, the murine back abscess model is used to study the host response to the polymicrobial mixed infection, including *P.gingivalis, A.actinomycetecommitans,* and *F nucleatum* (57). Owing to the genetic variances among the strains affecting the mutation of the immune components or in their adaptive immunity, these strains are differentially susceptible to experimental periodontal diseases, and several strains are quite resistant. BALB/c, AKR/J, DBA/2J and C3H/HeN mice are more susceptible than C57BL6 A/J 129/J, SJL/J and C3H/HeJ (58, 59). Recently, a research group successfully generated a chronic periodontitis model in mice by oral infection of the *P. gingivalis* P4 strain, which co-aggregated with a dominant mouse oral commensal bacterium (60). A recent study experiments on humans and mice model demonstrated that TH17 cells are necessary for the breakdown of periodontal tissue (40). The murine ligature-induced periodontitis (LIP) model is frequently employed to study the dysbiosis of the indigenous oral microbiome, which has coevolved with the murine host microbiome in periodontitis (61, 62). Arce et al. compared the microbial profiles from 16S rDNA gene sequencing datasets from nine different murine LIP models on C57/BL6 mice and demonstrated that though there were similar enrichment of certain microbes across different studies, the organization of the microbial community was influenced by the induction of periodontitis and the specific study being conducted (61). They also illustrated the technical factors that influence the results of oral microbiome research in the periodontitis models, ranging from sample collection to bioinformatic pipeline, which has been emphasized previously (63).

### 5.3 Oral cancer

One of the earlier studies to demonstrate the role of oral microbes, precisely two periodontal pathogens in oral tumor progression, utilized male BALB/c mice models (64). Germ-free Swiss Webster male mice under gnotobiotic conditions were used to compare the tumourigenesis between OSCC-associated microbiomes after 4-nitroquinoline-1-oxide (4-NQO) induction (65). A recent study explored the impact of the microbiome and its metabolic pathways on regulating oral carcinogenesis using C57BL/6 mice and identified an increase in the biosynthesis of polyamines in saliva with oral cancer (66). Another study utilised BALB/c mice to explore the microbiome of OSCC in mice with experimental induced periodontitis where they identified *Porphyromonas* to be the most abundant genus (67). However, the impact of the variation in oral microbiome between the strains has not been taken into consideration in any of these studies. Given the significant differences between mice strains, Gnotobiotic or germ-free mice, Specific Pathogen-Free (SPF) Mice, Immunodeficient Mouse Models such as nonobese diabetic/severe combined immunodeficiency (NOD–SCID) or NOD SCID gamma (NSG) mice, Transgenic or Knockout Mice could be valuable for introducing specific microbial communities to study their effects on oral cancer development. Apart from rats and mice, male Golden Syrian hamsters (3–4 weeks) were also utilized to study the impact of smokeless tobacco on the oral microbiota and oral carcinogenesis (68).

The murine oral cavity is also ideal for the candida model and thus may serve perfectly for experimentation for clarification of the controversial role of candidal infection in OPMDs and OSCC. Various researchers have induced Candidiasis in germ-free mice (69, 70). In hyposalivation rat models (salivary glands were removed), it was demonstrated that disease progression was noted with more ALS transcripts (cell surface glycoprotein associated with adhesion) (69). Also, similar expression was noted in HIV-positive patients and experimental candidiasis in rats (70).

## 6 Gnotobiotic and germ-free rodents in oral microbiome research

In recent years, gnotobiotic animal models have emerged as a crucial tool for investigating intricate interactions between hosts and their microbiota. Gnotobiotic animal models consist of germ-free animals, devoid of any foreign organisms, and animals colonized with one or more specific microbes (71, 72). Germ-free mice are selectively bred in isolators that exclude entirely any contact with microorganisms, with the specific purpose of ensuring their complete absence of detectable bacteria, viruses, and eukaryotic germs (73). Germ-free rodents have no microorganisms living in or on them, allowing researchers to precisely control an animal's oral microbiome through the direct inoculation of bacteria of interest, which facilitates the exploration of intricate host-microbe interactions within the oral cavity. These models offer valuable insights into the complexities of the oral microbial ecosystem and aid in the development of targeted therapeutic strategies for maintaining oral health. The idea of germ-free mouse colonies was first proposed by Louis Pasteur in 1885 and later established in the 1940s (74). Germ-free mice are used to investigate the complete absence of microorganisms or to create gnotobiotic animals that are only colonized by identified germs. Nevertheless, the production and upkeep of these mice necessitate specialized facilities, and the expenses, effort, and expertise needed to maintain them can render these models unattainable for several researchers (75). To ensure the absence of germs in mice, it is necessary to regularly check for contamination using various methods such as culture, microscopy, serology, gross morphology, and sequencing-based detection approaches (76). They also utilize PCR (both 16S and pathogen-specific), microscopy, and culturing techniques to test for bacteria. In addition, any distinct mouse strain to be examined in germ-free circumstances must be regenerated in these facilities, which restricts the number of diverse genotypes that may be feasibly studied (77). In addition, the upkeep of mice in isolators may render it unfeasible or arduous to carry out specific investigations, such as behavioral assessments or pathogen introductions. However, it is essential to differentiate this term from specified pathogen-free (SPF), as SPF animals are devoid of specific pathogens. Still, their internal microbiota is intricate and not clearly characterized (78). Gnotobiotic mouse models contribute significantly to our understanding of the oral microbiome's role in health and disease, enabling the investigation of dysbiosis, immune responses, and microbial contributions to conditions such as dental caries, periodontal diseases, and oral cancer.

## 7 Humanization strategies in germ-free mice for oral microbiome

Humanization strategies for oral microbiome research in germ-free mice involve introducing human oral microbial communities into mice raised in a germ-free environment. The resemblance of the transplanted oral microbiome in mice to that of the donor, i.e., the human microbiome, is dependent upon several factors like diet, socioeconomic background, ethnicity, exercise regime, etc. Differences in the donor and recipient anatomy and genetic, environmental, and immunological backgrounds are the key factors affecting this similarity. Human microbiome transplantation allows the oral samples from human donors to be transplanted into germ-free mice. Fecal Microbiota Transplantation (FMT), although primarily associated with the gut microbiome, has also been adapted for oral microbiome research (79). Human oral microbial communities extracted from fecal samples are introduced into germ-free mice. He et al. have transplanted the microbiome of periodontitis patients into the mouse periodontium and observed a significant increase in osteoclasts, TNF-α, and IL-1β in the latter (80). Human periodontitis microbiome has also been introduced in mouse by using subgingival plaque of periodontitis patients (81). However, several host factors could have a significant effect on microbial colonization (82). Another significant advantage would be the immunological response elicited by these humanized oral bacteria communities. Immunodeficient mice may be studied, but they will once more be unable to mimic the reaction that oral microbiological communities will have in a human setting. Despite limitations and challenges, humanized mice models hold promise in elucidating the pathogenesis and immunotherapeutic strategies in periodontitis. There have been notable findings from studies using humanized models. *A. actinomycetemcomitans*-specific IgG antibody responses, significant increment in the RANKL expression and alveolar bone resorption, as well as decreased OPG expression, was observed in the Aa-hu-PBL-NOD/SCID model (83). These models also shed light on the role of suppressor of cytokine signaling (SOCS) molecules *in A. actinomycetemcomitans*-induced osteoclastogenesis. By using a *P. gingivalis* infected HLA-DR1 humanized C57BL/6 mice model, the development of rheumatoid arthritis and its impact on bone density and systemic cytokine production were also analyzed (84). Recently, a team of researchers developed a human oral microbiota-associated (HOMA) mouse model by transplanting human saliva into germ-free mice and 85% of the genus-level taxa were identified to be similar to the donor (16).

## 8 Conclusion

Although the comparative physiology of rodents and humans emphasizes the significance of rodents as a model to study oral pathologies, the literature unequivocally demonstrates that genetics plays a significant role in oral microbiome variability among rodents. Diet and living conditions are also significant variables that must be considered, particularly when replicating human microbial diseases and comparing the findings of oral microbiome studies. Future research should prioritize replicating microbial studies with different rodent strains to demonstrate strong and reliable results regardless of diet, genotype, or environmental factors on the composition of the oral microbiome. Exploring germ-free mice and then enhancing humanization strategies would be a way forward. Given the significant differences between the human oral microbiota and that of mice, it is essential to conduct thorough robustness checks before extrapolating the results to humans.

## References

[1] Bracken MB. Why animal studies are often poor predictors of human reactions to exposure. J R Soc Med. (2009) 102(3):120–2. 10.1258/jrsm.2008.08k033 19297654 PMC2746847

[2] Di Cosola M Cazzolla AP Charitos IA Ballini A Inchingolo F Santacroce L. Candida albicans and oral carcinogenesis. A brief review. J Fungi (Basel). (2021) 7(6):476. 10.3390/jof7060476 34204731 PMC8231483

[3] Muller S Tilakaratne WM. Update from the 5th edition of the world health organization classification of head and neck tumors: tumours of the oral cavity and mobile tongue. Head Neck Pathol. (2022) 16(1):54–62. 10.1007/s12105-021-01402-9 35312982 PMC9018914

[4] Su Mun L Wye Lum S Kong Yuiin Sze G Hock Yoong C Ching Yung K Kah Lok L Association of microbiome with oral squamous cell carcinoma: a systematic review of the metagenomic studies. IJERPH. (2021) 18(14):7224. 10.3390/ijerph18147224 34299675 PMC8306663

[5] Meisel P Holtfreter B Biffar R Suemnig W Kocher T. Association of periodontitis with the risk of oral leukoplakia. Oral Oncol. (2012) 48(9):859–63. 10.1016/j.oraloncology.2012.02.022 22436883

[6] Gopinath D Kunnath Menon R Veettil SK George Botelho M Johnson NW. Periodontal diseases as putative risk factors for head and neck cancer: systematic review and meta-analysis. Cancers (Basel). (2020) 12(7):1893. 10.3390/cancers12071893 32674369 PMC7409086

[7] Hufeldt MR Nielsen DS Vogensen FK Midtvedt T Hansen AK. Variation in the gut microbiota of laboratory mice is related to both genetic and environmental factors. Comp Med. (2010) 60(5):336–47. 21262117 PMC2958200

[8] Khayatan D Hussain A Tebyaniyan H. Exploring animal models in oral cancer research and clinical intervention: a critical review. Vet Med Sci. (2023) 9(4):1833–47. 10.1002/vms3.1161 37196179 PMC10357283

[9] Mancinelli E Capello V. Anatomy and disorders of the oral cavity of rat-like and squirrel-like rodents. Vet Clin North Am Exot Anim Pract. (2016) 19(3):871–900. 10.1016/j.cvex.2016.04.008 27497210 PMC7110795

[10] Cox PG Jeffery N. Reviewing the morphology of the jaw-closing musculature in squirrels, rats, and guinea pigs with contrast-enhanced MicroCt. Anat Rec. (2011) 294(6):915–28. 10.1002/ar.21381 21538924

[11] Cox PG Rayfield EJ Fagan MJ Herrel A Pataky TC Jeffery N. Functional evolution of the feeding system in rodents. PLoS One. (2012) 7(4):e36299. Goswami A, editor. 10.1371/journal.pone.0036299 22558427 PMC3338682

[12] Takeda M Hoshino T. Fine structure of taste buds in the rat. Arch Histol Jpn. (1975) 37(5):395–413. 10.1679/aohc1950.37.395 143255

[13] Dewhirst FE Chen T Izard J Paster BJ Tanner ACR Yu WH The human oral microbiome. J Bacteriol. (2010) 192(19):5002–17. 10.1128/JB.00542-10 20656903 PMC2944498

[14] Abusleme L O’Gorman H Dutzan N Greenwell-Wild T Moutsopoulos NM. Establishment and stability of the murine oral microbiome. J Dent Res. (2020) 99(6):721–9. 10.1177/0022034520915485 32345105 PMC7243417

[15] Chun J Kim KY Lee JH Choi Y. The analysis of oral microbial communities of wild-type and toll-like receptor 2-deficient mice using a 454 GS FLX titanium pyrosequencer. BMC Microbiol. (2010) 10(1):101. 10.1186/1471-2180-10-101 20370919 PMC2873484

[16] Li B Ge Y Cheng L Zeng B Yu J Peng X Oral bacteria colonize and compete with gut microbiota in gnotobiotic mice. Int J Oral Sci. (2019) 11(1):10. 10.1038/s41368-018-0043-9 30833566 PMC6399334

[17] Coulombe C Lavoie MC. Evolution of resident oral bacterial biota in BALB/c mice during pregnancy and lactation. Microb Ecol. (1995) 30(2):219–25. 10.1007/BF00172576 24185487

[18] Chung MK Lee J Duraes G Ro JY. Lipopolysaccharide-induced pulpitis up-regulates TRPV1 in trigeminal ganglia. J Dent Res. (2011) 90(9):1103–7. 10.1177/0022034511413284 21712529 PMC3169884

[19] Sellers RS Clifford CB Treuting PM Brayton C. Immunological variation between inbred laboratory mouse strains: points to consider in phenotyping genetically immunomodified mice. Vet Pathol. (2012) 49(1):32–43. 10.1177/0300985811429314 22135019

[20] Yalcin B Wong K Agam A Goodson M Keane TM Gan X Sequence-based characterization of structural variation in the mouse genome. Nature. (2011) 477(7364):326–9. 10.1038/nature10432 21921916 PMC3428933

[21] Joseph S Aduse-Opoku J Hashim A Hanski E Streich R Knowles SCL A 16S rRNA gene and draft genome database for the murine oral bacterial community. mSystems. (2021) 6(1):e01222–20. Segata N, editor. 10.1128/msystems.01222-20 33563782 PMC7883545

[22] Hildebrand F Nguyen TLA Brinkman B Yunta R Cauwe B Vandenabeele P Inflammation-associated enterotypes, host genotype, cage and inter-individual effects drive gut microbiota variation in common laboratory mice. Genome Biol. (2013) 14(1):R4. 10.1186/gb-2013-14-1-r4 23347395 PMC4053703

[23] Kovacs A Ben-Jacob N Tayem H Halperin E Iraqi FA Gophna U. Genotype is a stronger determinant than sex of the mouse gut microbiota. Microb Ecol. (2011) 61(2):423–8. 10.1007/s00248-010-9787-2 21181142

[24] Long LL Svenson KL Mourino AJ Michaud M Fahey JR Waterman L Shared and distinctive features of the gut microbiome of C57BL/6 mice from different vendors and production sites, and in response to a new vivarium. Lab Anim (NY). (2021) 50(7):185–95. 10.1038/s41684-021-00777-0 34127866

[25] Sedghi L Byron C Jennings R Chlipala GE Green SJ Silo-Suh L. Effect of dietary fiber on the composition of the murine dental microbiome. Dent J. (2019) 7(2):58. 10.3390/dj7020058 31159370 PMC6630570

[26] Blais JF Lavoie MC. Effect of dietary components on the indigenous oral bacterial Flora of BALB/c mice. J Dent Res. (1990) 69(3):868–73. 10.1177/00220345900690030801 2324350

[27] Lai PS Allen JG Hutchinson DS Ajami NJ Petrosino JF Winters T Impact of environmental microbiota on human microbiota of workers in academic mouse research facilities: an observational study. PLoS One. (2017) 12(7):e0180969. Fehrenbach H, editor. 10.1371/journal.pone.0180969 28704437 PMC5509249

[28] Fischer AW Cannon B Nedergaard J. Optimal housing temperatures for mice to mimic the thermal environment of humans: an experimental study. Mol Metab. (2018) 7:161–70. 10.1016/j.molmet.2017.10.009 29122558 PMC5784327

[29] Toth LA Trammell RA Ilsley-Woods M. Interactions between housing density and ambient temperature in the cage environment: effects on mouse physiology and behavior. J Am Assoc Lab Anim Sci. (2015) 54(6):708–17. 26632780 PMC4671786

[30] Bär J Leung JM Hansen C Loke P Hall AR Conour L Strong effects of lab-to-field environmental transitions on the bacterial intestinal microbiota of *Mus musculus* are modulated by *Trichuris muris* infection. FEMS Microbiol Ecol. (2020) 96(10):fiaa167. 10.1093/femsec/fiaa167 32816007

[31] Rausch P Basic M Batra A Bischoff SC Blaut M Clavel T Analysis of factors contributing to variation in the C57BL/6J fecal microbiota across German animal facilities. Int J Med Microbiol. (2016) 306(5):343–55. 10.1016/j.ijmm.2016.03.004 27053239

[32] Roman LJ Snijders AM Chang H Mao JH Jones KJ Lawson GW. Effect of husbandry practices on the fecal microbiota of C57BL/6J breeding colonies housed in 2 different barrier facilities in the same institution. J Am Assoc Lab Anim Sci. (2023) 62(1):26–37. 10.30802/AALAS-JAALAS-22-000068 36755206 PMC9936858

[33] Menon RK Gomez A Brandt BW Leung YY Gopinath D Watt RM Long-term impact of oral surgery with or without amoxicillin on the oral microbiome-A prospective cohort study. Sci Rep. (2019) 9(1):18761. 10.1038/s41598-019-55056-3 31822712 PMC6904678

[34] Bailey MT Dowd SE Galley JD Hufnagle AR Allen RG Lyte M. Exposure to a social stressor alters the structure of the intestinal microbiota: implications for stressor-induced immunomodulation. Brain Behav Immun. (2011) 25(3):397–407. 10.1016/j.bbi.2010.10.023 21040780 PMC3039072

[35] Yang C Fujita Y Ren Q Ma M Dong C Hashimoto K. Bifidobacterium in the gut microbiota confer resilience to chronic social defeat stress in mice. Sci Rep. (2017) 7(1):45942. 10.1038/srep45942 28368029 PMC5377462

[36] Bangsgaard Bendtsen KM Krych L Sørensen DB Pang W Nielsen DS Josefsen K Gut microbiota composition is correlated to grid floor induced stress and behavior in the BALB/c mouse. PLoS One. (2012) 7(10):e46231. Aziz RK, editor. 10.1371/journal.pone.0046231 23056268 PMC3462757

[37] Galley JD Mackos AR Varaljay VA Bailey MT. Stressor exposure has prolonged effects on colonic microbial community structure in Citrobacter rodentium-challenged mice. Sci Rep. (2017) 7(1):45012. 10.1038/srep45012 28344333 PMC5366811

[38] Duran-Pinedo AE Solbiati J Frias-Lopez J. The effect of the stress hormone cortisol on the metatranscriptome of the oral microbiome. NPJ Biofilms Microbiomes. (2018) 4(1):25. 10.1038/s41522-018-0068-z 30345066 PMC6194028

[39] Rosshart SP Herz J Vassallo BG Hunter A Wall MK Badger JH Laboratory mice born to wild mice have natural microbiota and model human immune responses. Science. (2019) 365(6452):eaaw4361. 10.1126/science.aaw4361 31371577 PMC7377314

[40] Dutzan N Kajikawa T Abusleme L Greenwell-Wild T Zuazo CE Ikeuchi T A dysbiotic microbiome triggers T _H_ 17 cells to mediate oral mucosal immunopathology in mice and humans. Sci Transl Med. (2018) 10(463):eaat0797. 10.1126/scitranslmed.aat0797 30333238 PMC6330016

[41] Zaura E Brandt BW Teixeira de Mattos MJ Buijs MJ Caspers MP Rashid MU Same exposure but two radically different responses to antibiotics: resilience of the salivary microbiome versus long–term microbial shifts in feces. mBio. (2015) 6(6):e01693–15. 10.1128/mBio.01693-15 26556275 PMC4659469

[42] Bowen WH. Rodent model in caries research. Odontology. (2013) 101(1):9–14. 10.1007/s10266-012-0091-0 23129523

[43] Hsiao J Wang Y Zheng L Liu R Said R Hadjiyski L *In vivo* rodent models for studying dental caries and pulp disease. Methods Mol Biol. (2019) 1922:393–403. 10.1007/978-1-4939-9012-2_35 30838593

[44] Ohshima K Ohshima T Meyer K Takai E Yoshizawa S Shiraki K Proteome analysis of high affinity mouse saliva proteins to hydroxyapatite. Heliyon. (2022) 8(8):e10077. 10.1016/j.heliyon.2022.e10077 36033281 PMC9399162

[45] Renard E Gaudin A Bienvenu G Amiaud J Farges JC Cuturi MC Immune cells and molecular networks in experimentally induced pulpitis. J Dent Res. (2016) 95(2):196–205. 10.1177/0022034515612086 26472753

[46] Wu H Zeng B Li B Ren B Zhao J Li M Research on oral microbiota of monozygotic twins with discordant caries experience—*in vitro* and *in vivo* study. Sci Rep. (2018) 8(1):7267. 10.1038/s41598-020-61785-7 29740156 PMC5940813

[47] Tani N Kuchiba K Osada T Watanabe Y Umemoto T. Effect of T-cell deficiency on the formation of periapical lesions in mice: histological comparison between periapical lesion formation in BALB/c and BALB/c nu/nu mice. J Endod. (1995) 21(4):195–9. 10.1016/s0099-2399(06)80565-0 7673820

[48] da Silva RAB Ferreira PDF De Rossi A Nelson-Filho P Silva LAB. Toll-like receptor 2 knockout mice showed increased periapical lesion size and osteoclast number. J Endod. (2012) 38(6):803–13. 10.1016/j.joen.2012.03.017 22595116

[49] Bezerra da Silva RA Nelson-Filho P Lucisano MP De Rossi A de Queiroz AM Bezerra da Silva LA. Myd88 knockout mice develop initial enlarged periapical lesions with increased numbers of neutrophils. Int Endod J. (2014) 47(7):675–86. 10.1111/iej.12204 24127866

[50] Gan G Lin S Luo Y Zeng Y Lu B Zhang R Unveiling the oral-gut connection: chronic apical periodontitis accelerates atherosclerosis via gut microbiota dysbiosis and altered metabolites in apoE−/− mice on a high-fat diet. Int J Oral Sci. (2024) 16(1):39. 10.1038/s41368-024-00301-3 38740741 PMC11091127

[51] Haraga H Sato T Watanabe K Hamada N Tani-Ishii N. Effect of the progression of Fusobacterium nucleatum-induced apical periodontitis on the gut microbiota. J Endod. (2022) 48(8):1038–45. 10.1016/j.joen.2022.04.014 35545147

[52] Monasterio G Castillo F Ibarra JP Guevara J Rojas L Alvarez C Alveolar bone resorption and Th1/Th17-associated immune response triggered during *Aggregatibacter actinomycetemcomitans* -induced experimental periodontitis are serotype-dependent. J Periodontol. (2018) 89(10):1249–61. 10.1002/JPER.17-0563 30030845

[53] Monasterio G Budini V Fernández B Castillo F Rojas C Alvarez C IL -22–expressing CD 4 ^+^ AhR ^+^ T lymphocytes are associated with RANKL -mediated alveolar bone resorption during experimental periodontitis. J Periodontal Res. (2019) 54(5):513–24. 10.1111/jre.12654 31032952

[54] Acqua YD Hernández C Fogacci M Barbirato D Palioto D. Local and systemic effects produced in different models of experimental periodontitis in mice: a systematic review. Arch Oral Biol. (2022) 143:105528. 10.1016/j.archoralbio.2022.105528 36063643

[55] Klausen B. Microbiological and immunological aspects of experimental periodontal disease in rats: a review article. J Periodontol. (1991) 62(1):59–73. 10.1902/jop.1991.62.1.59 2002433

[56] Baker PJ Evans RT Roopenian DC. Oral infection with Porphyromonas gingivalis and induced alveolar bone loss in immunocompetent and severe combined immunodeficient mice. Arch Oral Biol. (1994) 39(12):1035–40. 10.1016/0003-9969(94)90055-8 7717884

[57] Oz HS Puleo DA. Animal models for periodontal disease. J Biomed Biotechnol. (2011) 2011:754857. 10.1155/2011/754857 21331345 PMC3038839

[58] Nakamura H Fukusaki Y Yoshimura A Shiraishi C Kishimoto M Kaneko T Lack of toll-like receptor 4 decreases lipopolysaccharide-induced bone resorption in C3H/HeJ mice *in vivo*. Oral Microbiol Immunol. (2008) 23(3):190–5. 10.1111/j.1399-302X.2007.00410.x 18402604

[59] Baker PJ Dixon M Roopenian DC. Genetic control of susceptibility to Porphyromonas gingivalis-induced alveolar bone loss in mice. Infect Immun. (2000) 68(10):5864–8. 10.1128/IAI.68.10.5864-5868.2000 10992496 PMC101548

[60] Liu M Choi Y. A murine periodontitis model using coaggregation between human pathogens and a predominant mouse oral commensal bacterium. J Periodontal Implant Sci. (2022) 52(2):141. 10.5051/jpis.2104360218 35505575 PMC9064776

[61] Arce M Endo N Dutzan N Abusleme L. A reappraisal of microbiome dysbiosis during experimental periodontitis. Mol Oral Microbiol. (2022) 37(5):180–95. 10.1111/omi.12382 35861180

[62] Abe T Hajishengallis G. Optimization of the ligature-induced periodontitis model in mice. J Immunol Methods. (2013) 394(1–2):49–54. 10.1016/j.jim.2013.05.002 23672778 PMC3707981

[63] Hernández-Arriaga A Baumann A Witte OW Frahm C Bergheim I Camarinha-Silva A. Changes in oral microbial ecology of C57BL/6 mice at different ages associated with sampling methodology. Microorganisms. (2019) 7(9):283. 10.3390/microorganisms7090283 31443509 PMC6780121

[64] Gallimidi AB Fischman S Revach B Bulvik R Maliutina A Rubinstein AM Periodontal pathogens *Porphyromonas gingivalis* and *Fusobacterium nucleatum* promote tumor progression in an oral-specific chemical carcinogenesis model. Oncotarget. (2015) 6(26):22613–23. 10.18632/oncotarget.4209 26158901 PMC4673186

[65] Stashenko P Yost S Choi Y Danciu T Chen T Yoganathan S The oral mouse microbiome promotes tumorigenesis in oral squamous cell carcinoma. mSystems. (2019) 4(4):e00323–19. 10.1128/mSystems.00323-19 31387932 PMC6687944

[66] Chen YL Huang KC Wu JH Liu T Chen JW Xie JY Microbiome dysbiosis inhibits carcinogen-induced murine oral tumorigenesis. J Cancer. (2022) 13(10):3051–60. 10.7150/jca.75947 36046649 PMC9414028

[67] Wei W Li J Shen X Lyu J Yan C Tang B Oral microbiota from periodontitis promote oral squamous cell carcinoma development via *γδ* T cell activation. mSystems. (2022) 7(5):e00469–22. Gilbert JA, editor. 10.1128/msystems.00469-22 36000726 PMC9600543

[68] Jin J Guo L VonTungeln L Vanlandingham M Cerniglia CE Chen H. Smokeless tobacco impacts oral microbiota in a Syrian golden hamster cheek pouch carcinogenesis model. Anaerobe. (2018) 52:29–42. 10.1016/j.anaerobe.2018.05.010 29852249 PMC6109425

[69] Costa AC Pereira CA Junqueira JC Jorge AO. Recent mouse and rat methods for the study of experimental oral candidiasis. Virulence. (2013) 4(5):391–9. 10.4161/viru.25199 23715031 PMC3714131

[70] Green CB Marretta SM Cheng G Faddoul FF Ehrhart EJ Hoyer LL. RT–PCR analysis of *Candida albicans* ALS gene expression in a hyposalivatory rat model of oral candidiasis and in HIV-positive human patients. Med Mycol. (2006) 44(2):103–11. 10.1080/13693780500086527 16519012 PMC2583129

[71] Basic M Bleich A. Gnotobiotics: past, present and future. Lab Anim. (2019) 53(3):232–43. 10.1177/0023677219836715 31096878

[72] Al-Asmakh M Zadjali F. Use of germ-free animal models in microbiota-related research. J Microbiol Biotechnol. (2015) 25(10):1583–8. 10.4014/jmb.1501.01039 26032361

[73] Kennedy EA King KY Baldridge MT. Mouse microbiota models: comparing germ-free mice and antibiotics treatment as tools for modifying gut bacteria. Front Physiol. (2018) 9:1534. 10.3389/fphys.2018.01534 30429801 PMC6220354

[74] Yi P Li L. The germfree murine animal: an important animal model for research on the relationship between gut microbiota and the host. Vet Microbiol. (2012) 157(1–2):1–7. 10.1016/j.vetmic.2011.10.024 22079217

[75] Nicklas W Keubler L Bleich A. Maintaining and monitoring the defined microbiota status of gnotobiotic rodents. ILAR J. (2015) 56(2):241–9. 10.1093/ilar/ilv029 26323633

[76] Fontaine CA Skorupski AM Vowles CJ Anderson NE Poe SA Eaton KA. How free of germs is germ-free? Detection of bacterial contamination in a germ free mouse unit. Gut Microbes. (2015) 6(4):225–33. 10.1080/19490976.2015.1054596 26018301 PMC4615677

[77] Daoust L Choi BSY Agrinier AL Varin TV Ouellette A Mitchell PL Gnotobiotic mice housing conditions critically influence the phenotype associated with transfer of faecal microbiota in a context of obesity. Gut. (2023) 72(5):896–905. 10.1136/gutjnl-2021-326475 36881441

[78] Dobson GP Letson HL Biros E Morris J. Specific pathogen-free (SPF) animal status as a variable in biomedical research: have we come full circle? EBioMedicine. (2019) 41:42–3. 10.1016/j.ebiom.2019.02.038 30803932 PMC6443024

[79] Nascimento MM. Oral microbiota transplant: a potential new therapy for oral diseases. J Calif Dent Assoc. (2017) 45(10):565–8. 29497269 PMC5828680

[80] He J Shen X Fu D Yang Y Xiong K Zhao L Human periodontitis-associated salivary microbiome affects the immune response of diabetic mice. J Oral Microbiol. (2022) 14(1):2107814. 10.1080/20002297.2022.2107814 35958276 PMC9359160

[81] Bai L Chen BY Liu Y Zhang WC Duan SZ. A mouse periodontitis model with humanized oral bacterial community. Front Cell Infect Microbiol. (2022) 12:842845. 10.3389/fcimb.2022.842845 35273925 PMC8902145

[82] Wu YY Westwater C Xiao E Dias Corrêa J Xiao WM Graves DT. Establishment of oral bacterial communities in germ-free mice and the influence of recipient age. Mol Oral Microbiol. (2018) 33(1):38–46. 10.1111/omi.12194 28776953 PMC6525632

[83] Zhang X Alnaeeli M Singh B Teng YTA. Involvement of SOCS3 in regulation of CD11c+ dendritic cell-derived osteoclastogenesis and severe alveolar bone loss. Infect Immun. (2009) 77(5):2000–9. 10.1128/IAI.01070-08 19255186 PMC2681769

[84] Sandal I Karydis A Luo J Prislovsky A Whittington KB Rosloniec EF Bone loss and aggravated autoimmune arthritis in HLA-DR*β*1-bearing humanized mice following oral challenge with Porphyromonas gingivalis. Arthritis Res Ther. (2016) 18(1):249. 10.1186/s13075-016-1143-6 27784339 PMC5081677

